# Skeletal outcomes of patients with osteogenesis imperfecta during drug holiday of bisphosphonates: a real-world study

**DOI:** 10.3389/fendo.2022.901925

**Published:** 2022-09-26

**Authors:** Yongze Zhang, Jing Hu, Xiaoyun Lin, Lei Sun, Sunjie Yan, Qian Zhang, Yan Jiang, Ou Wang, Weibo Xia, Xiaoping Xing, Mei Li

**Affiliations:** ^1^ Department of Endocrinology, Key Laboratory of Endocrinology of Ministry of Health, Peking Union Medical College Hospital, Peking Union Medical College, Chinese Academy of Medical Science, Beijing, China; ^2^ Department of Endocrinology, the First Affiliated Hospital of Fujian Medical University, Fuzhou, China

**Keywords:** skeletal outcomes, bisphosphonate, drug holiday, osteogenesis imperfecta, long-term therapy

## Abstract

**Purpose:**

This study aimed to investigate the skeletal outcomes of patients with osteogenesis imperfecta (OI) who received bisphosphonate (BP) treatment and entered drug holiday after achieving an age- and sex-specific bone mineral density (BMD) reference.

**Methods:**

Patients with OI receiving BP treatment were enrolled when they entered drug holidays of BPs. The skeletal outcomes were evaluated in detail during the drug holiday, including BMD, X-ray of the bone, bone fracture incidence, and bone turnover biomarkers. The pathogenic mutations of OI were identified by next-generation sequencing and confirmed by Sanger sequencing.

**Results:**

A total of 149 OI patients (127 juveniles and 22 adults) who entered drug holidays after nearly 4 years of BP treatment were included. Areal BMD at the lumbar spine increased from 0.934 ± 0.151 to 0.990 ± 0.142 g/cm^2^ and was stable in the second (1.029 ± 0.176 g/cm^2^) and third years (1.023 ± 0.174 g/cm^2^) of BP drug holidays, and BMD at the femoral neck, trochanter, and total hip had no significant change, but it was gradually inferior to that of the same-gender juveniles in the second and third years of the drug holiday. BMD at the lumbar spine and proximal hip did not change and was inferior to that of the same-gender adults. The average time of fractures fluctuated from 0.18 to 0.08 per year in juveniles, while only one adult suffered from a fracture during BP drug holidays. Bone turnover markers were in the normal range, except for a mildly high level of β-carboxy-terminal cross-linked telopeptide of type 1 collagen in the juvenile group. A total of 17 (11.4%) patients received BP retreatment because of bone loss during the drug holiday. OI type III and type IV and *COL1A2* mutation were correlated to a longer duration of BP treatment to enter drug holidays (all *p* < 0.05). Old age at initial treatment (OR, 1.056) and OI type III (OR, 10.880) were correlated to a higher risk of BP retreatment.

**Conclusions:**

OI patients will undergo nearly 4 years of BP treatment to achieve drug holidays. During the 3 years of the drug holiday, the patients’ BMD is stable, and fracture incidence does not increase significantly. Patients are more inclined to need retreatment during drug holidays owing to the late start of BP treatment and more severe OI phenotypes.

## Introduction

Osteogenesis imperfecta (OI) is an inherited skeletal dysplasia characterized by bone fragility and skeletal deformities, with an estimated incidence of 1 in 10,000–20,000 neonates (1, 2). OI also leads to dental and craniofacial abnormalities, muscle weakness, hearing loss, and respiratory and cardiovascular complications (1). The majority of OI patients are associated with pathogenic variants in *COL1A1* and *COL1A2*, the encoding genes of type I collagen, and the minority of OI patients are related to mutations in other genes that are involved in type I collagen biosynthesis or osteoblast differentiation or bone mineralization (3). The clinical classification of OI includes type I to type V, of which the severity broadly ranges from nearly asymptomatic cases with a normal life span to severe bone deformities, mobility impairment, and even perinatal mortality (4).

Treatment for OI is a great challenge, which is primarily supportive and symptomatic, including management with medications, physical therapy, and even orthopedic interventions to improve bone strength, reduce fracture risk, and improve mobility (5, 6). Bisphosphonates (BPs) are the most commonly used medications for OI, which can increase bone mineral density (BMD), reduce bone fracture risk, and lead to the reshaping of the vertebra (7–10), but the optimal duration of BP therapy in OI patients is unknown. Studies have shown that the therapeutic benefits for OI patients from BP treatment are more apparent in the first 2 to 4 years (11). Otherwise, iatrogenic osteopetrosis has been described with excessive treatment, and long-term BP therapy is associated with an increased risk of atypical femoral fracture in patients with OI (12, 13). Thus, many clinical concerns about BP treatment are worthy of investigation in OI patients, including when to enter the drug holiday of BPs, how long the drug holiday should hold, and how the skeletal outcomes during BP discontinuation.

Therefore, we aimed to investigate the skeletal outcomes during the drug holiday of BPs and their associated factors for OI patients when they entered the drug holiday.

## Materials and methods

### Study participants

Patients were diagnosed with OI if they had either a history of at least one non-traumatic or low-impact fracture and an age-adjusted and sex-adjusted areal BMD Z-score of –1.0 or less for either total body or lumbar spine sites, or an adjusted areal BMD Z-score of –2.0 or less irrespective of a history of fractures (14, 15). For patients without a family history of non-traumatic fracture, a diagnosis of OI was made if they had more than one non-traumatic fracture and at least a kind of extra-skeletal manifestations or with a genetic diagnosis of OI (14, 15).

The study comprised patients with OI who received BP treatment (alendronate or ibandronate or zoledronic acid) between the years 2003 and 2019 in the Endocrinology Department of Peking Union Medical College Hospital (PUMCH) and who discontinued BP treatment after achieving the age- and sex-specific normal BMD of juveniles (16, 17) and adults (18), termed drug holiday (19).

The study was approved by the ethics committee of PUMCH. Written informed consent was obtained from the patient or legal guardian of the patients before they participated in this study.

### Data collection

The medical history was collected in detail, including the age of onset; the clinical information of bone pain, bone fracture, and bone deformity; and a family history of OI. The bone, joint, sclera, ears, and teeth were examined carefully. Detailed information about fractures, including time of the initiation, site, degree of trauma, frequency, and radiological evidence of fracture, was collected. The frequency of clinical fracture was calculated as the number of clinical fractures/disease courses. Bone deformities were evaluated, including limb bending, thoracic deformity, scoliosis, and pelvic deformity (20). The height of the juvenile was measured using a Harpenden stadiometer (Seritex Inc., Farmingdale, NJ, USA) and adjusted to age- and sex-specific Z-scores on the basis of reference data from the Chinese National Centers for Disease Control and Prevention (21). For patients who were unable to stand, their height was replaced by a body length in a supine position. Serious events were observed, including new bone fractures, osteonecrosis of the jaw, and atypical femoral fracture. Delayed fracture healing was also recorded during the follow-up.

### Biochemical measurements

Blood samples were obtained after overnight fasting for at least 8 h. The serum concentrations of calcium (Ca), phosphate (P), total alkaline phosphatase (ALP), alanine aminotransferase (ALT), and creatinine (Cr) were measured using an automatic biochemical analyzer (ADVIA 1800, Siemens Inc., Munich, Germany). The serum levels of β-cross-linked C-telopeptide of type I collagen (β-CTX), N-terminal propeptide of type I precollagen (P1NP), 25-hydroxyvitamin D (25OHD), and intact parathyroid hormone (PTH) were measured with an automated electrochemiluminescence system (Roche Diagnostics, Basel, Switzerland). The biochemical measurements were completed in the central laboratory of PUMCH.

### Bone mineral density and radiographic assessments

The BMD at the lumbar spine, femoral neck, trochanter, and total hip was measured by dual-energy X-ray absorptiometry (Lunar Prodigy Advance, GE Healthcare, Chicago, IL, USA). The BMD phantom scan was performed daily and detected no significant machine drifts during the 5-year study. The areal BMD values were converted into age- and sex-specific Z-scores using reference data from previous studies (16–18). The radiologic views of the skull, spine, hip, and limb were performed by the radiologists of PUMCH.

### Detection of pathogenic mutation of osteogenesis imperfecta patients

The pathogenic mutations of OI were detected using a panel for next-generation sequencing (NGS) (Illumina HiSeq2000 platform, Illumina, Inc., San Diego, CA, USA), which covered 20 known candidate genes of OI (*COL1A1*, *COL1A2*, *IFITM5*, *SERPINF1*, *FKBP10*, *CRTAP*, *P3H1*, *PPIB*, *SERPINH1*, *BMP1*, *PLOD2*, *SP7*, *TMEM38B*, *WNT1*, *CREB3H1*, *SPARC*, *PLS3*, *P4HB*, *SEC24D*, and *MBTPS2*). The experimental procedures followed a previously described protocol (15). The mutations identified by NGS were further confirmed by Sanger sequencing.

### Classification of osteogenesis imperfecta

Patients with OI were classified into subtypes based on Sillence classification and molecular diagnosis (22): type I, mild phenotype; type II, perinatally lethal; type III, a severe form with progressive deformity; type IV, with moderate severity; and type V, characterized by calcification of the forearm interosseous membrane, radial head dislocation, and hyperplastic callus formation (23). No patients with OI type II were included in this study because of perinatal death. According to molecular diagnosis, genetic mutations leading to an early stop codon or frameshift in *COL1A1* were regarded as the quantitative reduction group (haploinsufficiency). Mutations causing amino acid substitutions in the triple-helical domain of *COL1A1* or *COL1A2* were classified into the qualitative defect group. As the effects of splice site mutation were difficult to predict, we did not include splice site mutations in the analyses (15).

### Statistical analysis

Normally distributed data, such as BMD and height, were presented as mean ± standard deviation, while those with abnormal distribution were expressed as medians (interquartile ranges (IQRs)), or proportions. The differences among each time of BMD, height, and so on, during BP discontinuation were analyzed using a generalized linear mixed model. Multiple linear and binary logistic regression analyses were used to analyze related factors of the BP treatment course and restart BP treatment. A *p*-value of less than 0.05 indicated a statistically significant difference. The statistical analyses were performed using SPSS 22.0 (SPSS Inc., Chicago, IL, USA). Graphs were drawn using GraphPad Prism software version 6.0.

## Results

### Patients’ characteristics at bisphosphonate discontinuation

**Figure 1:**
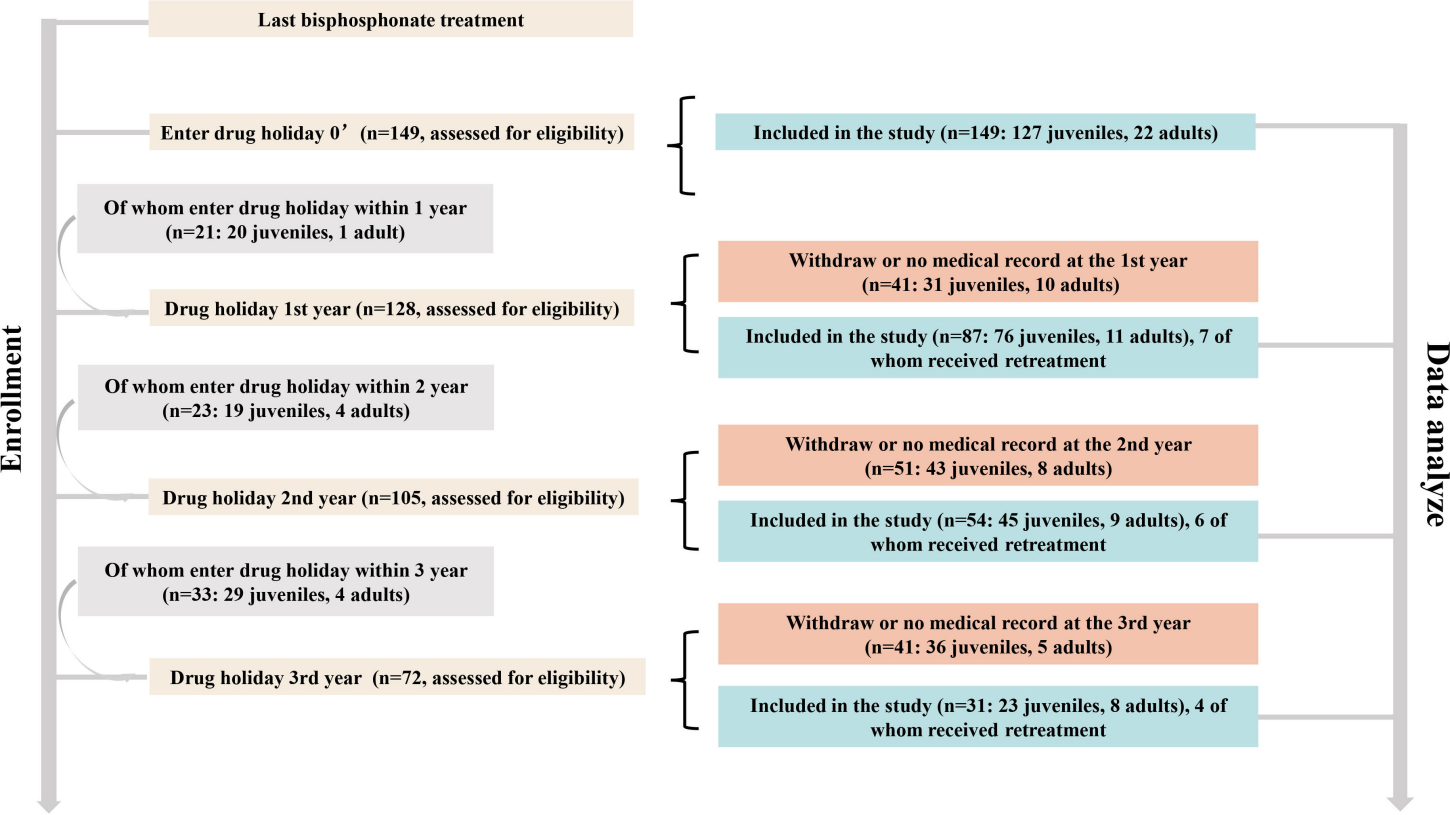
Details of the patients’ enrollment and the study design.

A total of 149 patients with OI received BP treatment and entered drug holiday, 127 of whom received BP treatment while they were still juveniles, designated as the juvenile group, while 22 patients received initial treatment during adulthood, designated as the adult group. These patients either received alendronate (12 in the juvenile group and 6 in the adult group) or zoledronate (95 in the juvenile group and 9 in the adult group) or were sequentially treated with alendronate or ibandronate and then with zoledronate (20 in the juvenile group and 7 in the adult group). The detailed follow-up records are shown in **Figure 1**. The median age of OI onset, age at initial treatment, and duration of BP treatment were 1.5 years (IQR 0.77–4.75 years), 7.0 years (IQR 3.1–11.8 years), and 3.95 years (IQR 2.2–5.0 years) in the juvenile group and were 2.0 years (IQR 1–11 years), 32.7 years (IQR 23.0–43.0 years), and 4.2 years (IQR 3.0–5.0 years) in the adult group, respectively, as shown in **Table 1**. Up to now, the time when OI patients entered drug holidays was as follows: 21 patients within 1 year, 23 patients within 2 years, 33 patients within 3 years, and 72 patients over 3 years (**Figure 1**). One patient had the longest follow-up of 7 years after BP discontinuation.

**Table 1:** Baseline characteristics of OI patients.

Characteristic	Juvenile group (n = 127)	Adult group (n = 22)
Male, n (%)	83 (65.4)	7 (31.8)
Age of onset, years, median (IQR)	1.5 (0.77–4.75)	2 (1–11)
Age at initial treatment, years, median (IQR)	7.0 (3.1–11.8)	32.6 (23.0–43.0)
Age of stopping BPs, years, median (IQR)	11.3 (7.7–15.8)	35.3 (27.6–46.8)
Duration of BP treatment, years, median (IQR)	3.95 (2.2–5.0)	4.2 (3.0–5.0)
Family history of fractures, n (%)	53 (41.7)	16 (72.7)
Family history of blue sclera, n (%)		
Father, n (%)	10 (7.8)	1 (4.5)
Mother, n (%)	9 (7.1)	3 (13.6)
Other consanguinity, n (%)	0 (0)	1 (4.5)
Fracture times, median (IQR)	3 (3)	5 (19)
Multiple fractures, n (%)	84 (66.1)	16 (72.7)
Difficulty walking, n (%)	32 (25.2)	4 (18.2)
Bone deformities, n (%)	35 (27.6)	6 (27.3)
Bone bending, n (%)	31 (24.4)	5 (22.7)
Loose joint, n (%)	62 (48.8)	11 (50)
Blue sclera, n (%)	103 (81.1)	17 (77.3)
Dentin deficiency, n (%)	23 (18.1)	5 (22.7)
Hearing impairment, n (%)	4 (3.1)	3 (13.6)
Medical Imaging		
Thin long bone cortex, n (%)	101 (79.5)	10 (45.5)
Interstitial bone, n (%)	75 (59.1)	9 (40.9)
Hypertrophic callus, n (%)	4 (3.1)	1 (4.5)
Sillence classification		
I, n (%)	80 (63)	17 (77.3)
III, n (%)	10 (7.9)	2 (9.1)
IV, n (%)	24 (18.9)	2 (9.1)
V, n (%)	13 (10.2)	1 (4.5)
Inheritance pattern		
Autosomal recessive inheritance, n (%)	38 (29.9)	8 (36.4)
Autosomal and X-linked dominant inheritance, n (%)	89 (70.1)^note^	14 (63.6)
Genetic mutations		
* COL1A1*, n (%)	54 (42.5)	6 (27.3)
* COL1A2*, n (%)	25 (19.7)	5 (22.7)
* IFITM5*, n (%)	8 (6.3)	1 (4.5)
* SERPINH1*, n (%)	3 (2.4)	0 (0)
*FKBP10*, n (%)	3 (2.4)	0 (0)
Others, n (%)	34 (26.8)	10 (45.5)
Collagen defects		
Quantitative reduction	22 (17.3)	6 (27.3)
Qualitative defect Unclassified	47 (37.0) 58 (45.7)	4 (18.2) 12 (54.5)

One child with OI inherited in an X-linked dominant way. BPs, bisphosphonates; OI, osteogenesis imperfecta; IQR, interquartile range.

The pathogenic variants of OI were identified in the majority of the patients. Sixty patients carried *COL1A1* mutation, 30 with *COL1A2* mutation, 9 with *IFITM5* mutation, 3 with *SERPINF1* mutation, 3 with *FKBP10* mutation, 2 with *PLOD2* mutation, and 2 with *WNT1* mutation, and mutations in *TMEM38B*, *CRTAP*, *PLS3*, and *P4HB* were found in one patient. Non-mutation was detected in 36 OI patients (**Table 1**).

### Skeletal outcomes after bisphosphonate discontinuation

**Figure 2:**
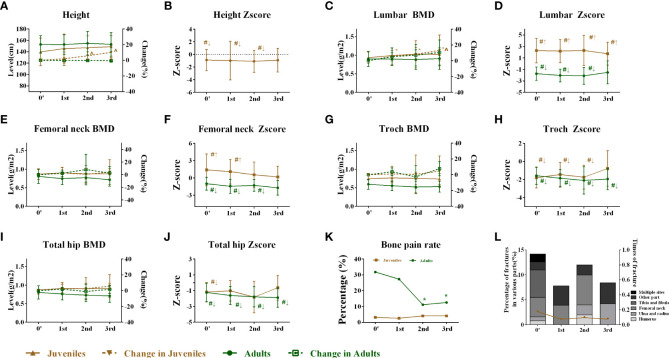
Changes in height and skeletal outcomes after bisphosphonate discontinuation. **(A)** Changes in height during the follow-up. **(B)** Changes in the height Z-score during the follow-up. **(C)** Changes in the lumbar BMD during the follow-up. **(D)** Changes in the lumbar Z-score during the follow-up. **(E)** Changes in the femoral neck BMD during the follow-up. **(F)** Changes in the femoral neck Z-score during the follow-up. **(G)** Changes in the trochanter BMD during the follow-up. **(H)** Changes in the trochanter Z-score during the follow-up. **(I)** Changes in the total hip BMD during the follow-up. **(J)** Changes in the total hip Z-score during the follow-up. **(K)** Changes in the bone pain rate during the follow-up. **(L)** Changes in average times per year of fractures and fracture rates during the follow-up. *Level compared with baseline after bisphosphonate discontinuation, *p* < 0.05. †Level compared with the first year after bisphosphonate discontinuation, *p* < 0.05. &Level compared with the second year after bisphosphonate discontinuation, *p* < 0.05. ^Change rate compared with 0′ after bisphosphonate discontinuation, *p* < 0.05. #Level compared with average Z-score of their peers (value = 0). ↑Superior to their same-sex peers. ↓Inferior to the same-sex peers. BMD, bone mineral density.

**Figure 3:**
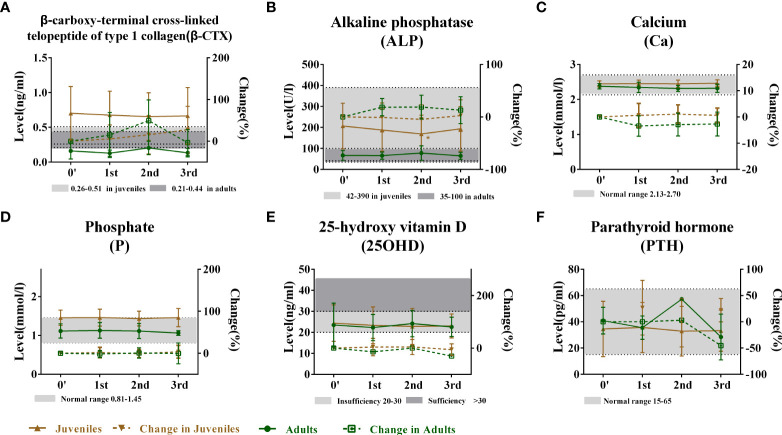
Changes in bone metabolic markers after bisphosphonate discontinuation. **(A)** Changes in the serum ALP level during the follow-up. Normal range of ALP (42–390 U/L in juvenile group and 35–100 U/L in adult group) marked by gray. **(B)** Changes in the serum β-CTX level during the follow-up. Normal range of β-CTX (0.26–0.51 ng/ml in juvenile group and 0.21–0.44 ng/ml in adult group) marked by gray. **(C)** Changes in serum Ca level during the follow-up. Normal range of Ca (2.13–2.7 mmol/L) marked by gray. **(D)** Changes in the serum P level during the follow-up. Normal range of P (0.81–1.45 mmol/L) marked by gray. **(E)** Changes in the serum 25OHD level during the follow-up. Level of 25OHD with more than 20 ng/ml marked by gray. **(F)** Changes in the serum PTH level during the follow-up. Normal range of PTH (15–65 pg/ml) marked by gray. *Level compared with baseline after bisphosphonate discontinuation, *p* < 0.05. †Level compared with the first year after bisphosphonate discontinuation, *p* < 0.05. &Level compared with the second year after bisphosphonate discontinuation, *p* < 0.05. ^Change rate compared with 0′ after bisphosphonate discontinuation, *p* < 0.05. ALP, alkaline phosphatase; PTH, parathyroid hormone.

After BP discontinuation, the height of juveniles with OI increased from 139.72 ± 24.28 to 145.51 ± 25.43, 147.05 ± 20.60, and 148.77 ± 19.38 cm in the first, second, and third years of drug holiday, respectively, but the juveniles had lower height Z-scores, which suggested that the OI patients were shorter than their peers. The heights of adults had no obvious change during the drug holidays (**Figures 2A, B**). Meanwhile, no significant change was observed in the serum levels of β-CTX, Ca, P, 25OHD, and PTH during the 3 years of the drug holidays in juveniles and adults, except for mildly increased β-CTX levels in juveniles (**Figures 3A–F**). Liver and kidney functions were normal during the 3 years of BP discontinuation (**Supplementary Figures 1A, B**). No patients suffered from osteonecrosis of the jaw and atypical femoral fracture during the whole drug holiday.

In juveniles, the areal BMD at the lumbar spine increased from 0.934 ± 0.151 to 0.990 ± 0.142 g/cm^2^ from the baseline (0′) to the first year of BP discontinuation and was stable in the second (1.029 ± 0.176 g/cm^2^) and third years (1.023 ± 0.174 g/cm^2^) in the juveniles, with no significant change in BMD Z-score (**Figures 2C, D**). The femoral neck BMD of juveniles was stable, but the Z-score gradually declined during the drug holidays (**Figures 2E, F**). No significant change was observed in the trochanter and the total hip BMD during the 3 years of BP discontinuation. However, the BMD of the juvenile was inferior to that of the peers in trochanter during the first and second years of the drug holidays (**Figures 2G–J**). The bone pain incidence fluctuated from 3.1% to 4.0%, and the average times of fractures of the juveniles fluctuated from 0.18 to 0.08 per year (**Figures 2K, L**).

In the adults, the BMD at the lumbar spine did not change in the 3 years of the drug holidays with a lower Z-score in the first 2 years until the third year (**Figures 2C, D**). The femoral neck BMD and Z-score of adults remained stable during the whole drug holiday (**Figures 2E, F**). Similarly, no significant change was observed in the trochanter and the total hip BMD during the 3 years of BP discontinuation. However, the BMD of adults was inferior to that of peers in trochanter during the 3 years of the drug holidays (**Figures 2G–J**). The bone pain incidence fluctuated from 31.8% to 12.5%, while only one adult suffered from a fracture in the second year of BP discontinuation.

### Related factors of bisphosphonate treatment course and restart treatment

**Figure 4:**
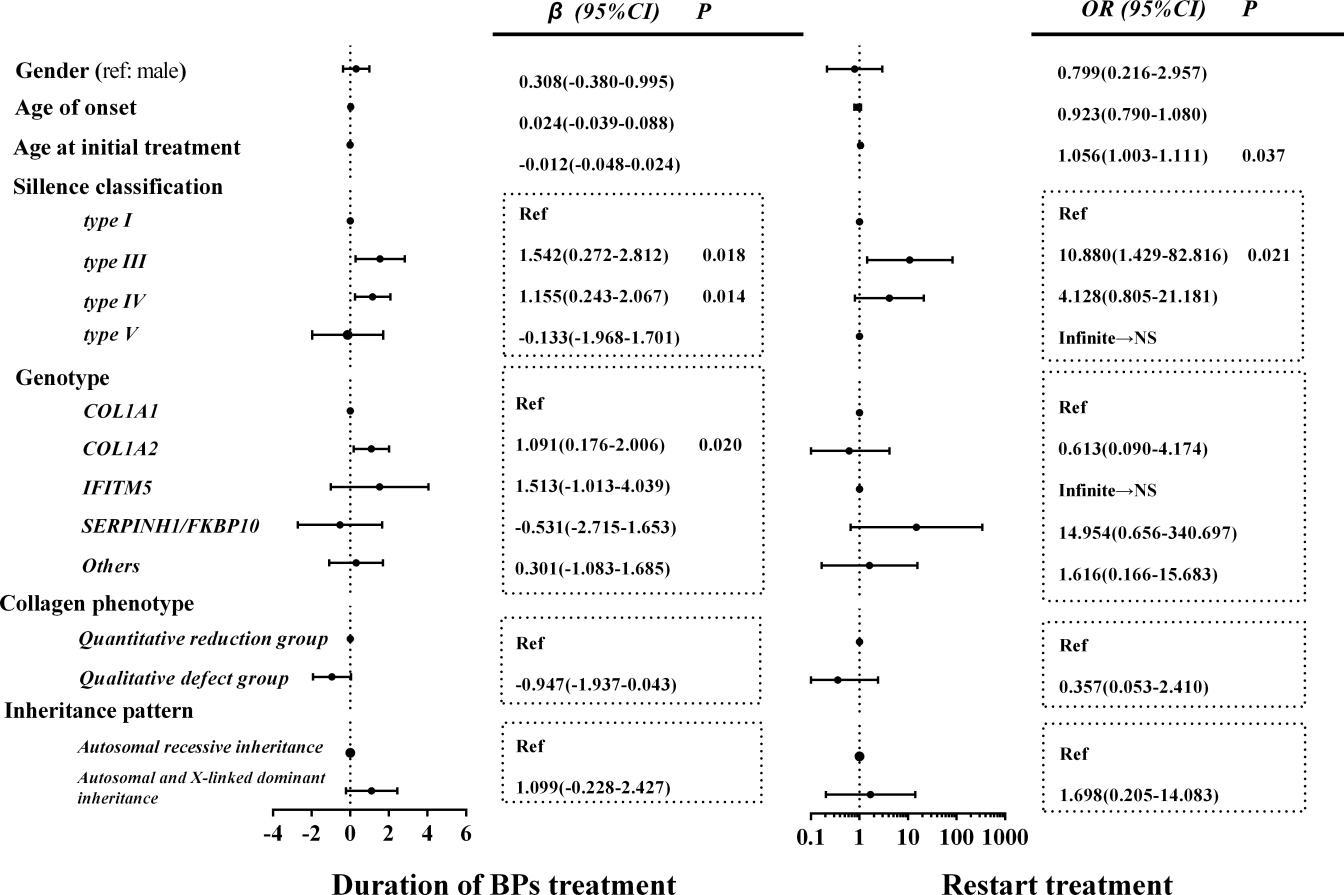
Related factors of BP treatment course and restart treatment of BPs. In the collagen phenotype group, those other than the quantitative and qualitative defect groups were classified as other groups to ensure that all data were fully analyzed. Data of other groups are not shown. NS, non-significant; BP, bisphosphonate.

We evaluated the related factors regarding the duration of BP treatment to enter the drug holiday. As shown in **Figure 4**, OI type III (β = 1.542, *p* = 0.018), OI type IV (β = 1.155, *p* = 0.014), and *COL1A2* mutation (β = 1.091, *p* = 0.020) were positively correlated with a longer duration of BP treatment before patients entered drug holiday.

There were 17 patients (11.4%, a median drug holiday of 2 years) who started the retreatment of BPs because of a decrease in BMD, including 12 juveniles (9.4%) and 5 adults (22.7%). The old age at initial treatment (OR, 1.056; 95% CI, 1.003–1.111) and type III OI (OR, 10.880; 95% CI, 1.429–82.816) were significantly correlated with the retreatment of BPs. However, no significant association was found between gender, age of OI onset, genotype, patterns of inheritance, and retreatment of BPs (**Figure 4**). The longest drug holiday was observed in an OI patient with a mutation in *COL1A2*, who received 2 years of zoledronic acid treatment and then entered a drug holiday. During the 7 years of the drug holiday, his lumbar and proximal hip BMD continued to increase (**Supplementary Table 1**). As he still had age- and sex-specific normal BMD, he did not receive retreatment.

## Discussion

This was a novel clinical study to evaluate the skeletal outcome during drug holidays of BPs in a large cohort of juveniles and adults with moderate-to-severe OI. During the 3-year observation in drug holidays, the serum levels of biochemical indexes were normal, except for a mildly high level of β-CTX in juveniles. The lumbar spine BMD increased in the first year and was stable in the second and third years of drug holiday in the juveniles. The proximal hip BMD had no significant change during the drug holiday, but its Z-score tended to decline gradually during the drug holidays in juveniles. Both lumbar spine and proximal hip BMD remained stable in the adult group. New fracture incidences remained at a lower level, which did not increase during the drug holiday. Meanwhile, OI type III and type IV and *COL1A2* mutation were positively correlated with a longer duration of BP treatment to achieve the drug holiday, while patients with a later onset of BP treatment and severe clinical phenotypes were associated with a higher risk of BP retreatment.

BPs are the most commonly used medications for osteoporosis, which play important roles by effectively inhibiting osteoclast activities, increasing BMD, and reducing bone fracture incidence (24). Nitrogenous BPs could disrupt osteoclasts formation, survival, and cytoskeletal dynamics, and alendronate, pamidronate, and zoledronate were commonly used BPs for patients with OI. However, long-term BP treatment could increase the microcracks in the bone, thereby increasing bone fragility and the risk of atypical fracture (25, 26). The persistent effects of BPs on the bone led to the concept of a drug holiday, which was designed to minimize side effects and maximize benefits. As the mechanical properties and structure of the bone in OI patients were markedly different from those of the normal bone, bone fragility was significantly high in OI patients. Therefore, we should consider the appropriate course of BP treatment in OI patients.

Recently, the optimal duration and long-term safety of BP therapy were worthy of further investigation. A meta-analysis showed that oral or intravenous BPs increased the BMD of children and adults with OI (27). However, whether oral or intravenous BP treatment could consistently reduce fracture occurrence was controversial (27). Although specific data were not extracted, BP therapy for 1 to 3 years appeared to be beneficial, with the maximum benefits in the first year of treatment in adults and children with OI patients (27, 28). The increase in load to fracture after BP treatment came at the cost of a trend toward a decline in bone material properties, decreased strength and elastic modulus, and decreased matrix production by osteoblasts, which could be avoided by a shorter treatment duration (29). In humans, the volume of the bone increased after BP treatment, while the intrinsic material properties, stiffness, and hardness of bone tissue remained unaffected (30). However, the average length of BP treatment preceding fractures was 6.5 years without standard drug holidays in the study by Nicolaou et al. (31). Thus, in another study, drug holidays were achieved after the average treatment duration of 4.1 years (7). In our study, a median of nearly 4.0 years of BP treatment was safe according to the skeletal outcomes. However, patients with OI are often not treated in a timely manner. OI is an extremely rare disease, and both doctors and patients might have relatively insufficient knowledge of disease treatment. As a result, the patients were diagnosed with OI but did not receive timely treatment until repeated fractures and even bone deformities occurred (32).

Additionally, when to terminate BP drug holiday and restart treatment were unclear in patients with OI. Rauch et al. suggested that treatment was restarted after 15 and 16 months of cessation of pamidronate when some patients began to feel unwell and lacked stamina (33). Another study indicated that BP treatment had to be restarted owing to the decreased BMD, increased fracture rate, and recurrence of bone pain (34). In our study, most patients with OI remained stable during the first 2 years of BP discontinuation. However, 17 (11.4%) patients received the retreatment after a median 2 years of drug holiday due to the obvious decrease in BMD.

Very few studies have comprehensively evaluated the skeletal outcome of OI patients during BP discontinuation. A study reported that the lumbar spine BMD and its *Z*-scores decreased, while the fracture rate increased in OI patients after 1.5 years of BP discontinuation (34). Another study indicated that the effects of pamidronate discontinuation were more obvious at the radial metaphysis than at the diaphysis (35). Recently, increased lumbar spine areal BMD and a 19% decrease in the trabecular volumetric BMD at the distal metaphysis were observed after 4 years of BP discontinuation in OI patients (36). Moreover, we evaluated the changes in bone turnover markers in juveniles and adults after BP discontinuation. BP discontinuation leads to an obvious decrease in bone turnover biomarkers. As OI children were in the stage of growth and development, juveniles with OI had increased BMD and slightly increased β-CTX levels during the BP drug holiday. Interestingly, the lumbar spine BMD continued to increase, and the proximal hip BMD had no significant change during the drug holidays in juveniles. The curative effects seemed to be weaker in OI adults than in OI juveniles, which may be related to high bone remodeling and bone growth speed in juveniles. The possible mechanism of site-specific changes in juveniles’ BMD was as follows: as the spine is rich in cancellous bone, the effects of BPs on the spine BMD were more obvious than in other sites (27, 37). BPs could not alter the genetic defects of OI, and the BMD would decrease again after the long-term discontinuation of BP therapy (33). The demineralization was predominant on sites rich in cortical bone (38), and a fall in BMD was significant in the proximal hip after BP discontinuation (39). Moreover, most studies focused on the safety profile of OI patients during treatment (40). No patients suffered from osteonecrosis of the jaw and atypical bone fracture in this study. Our findings might broaden the long-term safety spectrum of BPs in OI patients.

Several factors associated with the duration to enter a drug holiday and termination of the drug holidays were investigated. We found that OI type III and type IV and *COL1A2* mutation were positively correlated with a longer BP treatment duration to enter the drug holiday. Meanwhile, OI patients with later initiation of BP treatment or with the severe OI phenotype tended to require retreatment. As patients with OI type III or IV, or with a qualitative defect in type I collagen, usually had a severe phenotype, they might need a longer duration of BP treatment and need to be treated again (41, 42). As BPs were less effective in OI adults than in OI children (43, 44), the early initiation of BP treatment might have more benefits (40).

In this study, we comprehensively assessed the skeletal outcomes after BP discontinuation for the first time in a large cohort of Asian OI patients. We investigated the factors correlated with the BP treatment course to enter the drug holidays and retreatment during the drug holiday. Meanwhile, all the measurements were performed in a single center, avoiding measurement bias. However, this study had many limitations. It was a retrospective study. Patients were administered various BPs, and the duration of BP treatment and lengths of drug holidays were diverse.

## Conclusions

Patients with osteogenesis imperfecta will undergo nearly 4 years of BP treatment to achieve drug holidays. During the 3 years of the drug holiday, the patients’ BMD is stable, and fracture incidence does not increase significantly. Patients are more inclined to need retreatment during the drug holidays owing to the late start of BP treatment and more severe phenotypes This study provides valuable information for the long-term rational treatment of BPs in juveniles and adults with osteogenesis imperfecta.

## Data availability statement

The datasets for this article are not publicly available due to concerns regarding participant/patient anonymity. Requests to access the datasets should be directed to the corresponding authors. The datasets presented in this article are not readily available since no access to raw dataset of NGS is allowed other than the Beijing Genomics institution in charge of NGS. Requests to access the datasets should be directed to https://www.genomics.cn/contact.html.

## Ethics statement

The studies involving human participants were reviewed and approved by the ethics committee of PUMCH. Written informed consent to participate in this study was provided by the participants’ legal guardian/next of kin.

## Author contributions

YZ collected the clinical data from the patients, analyzed the data, and wrote the manuscript. JH, XL, and LS contributed to the data collection and blood sample collection. QZ, SY, YJ, OW, WX, and XX contributed to the review of the manuscript. ML contributed to the conception and design of the research, and acquisition and interpretation of the data, and revised the manuscript. All authors contributed to the article and approved the submitted version.

## Funding

This work is supported by National Key R&D Program of China (2018YFA0800801, 2021YFC2501704), CAMS Innovation Fund for Medical Sciences (CIFMS) (2021-I2M-C&T-B-007, 2021-I2M-1-051), National Natural Science Foundation of China (No. 81873668, 82070908), and Beijing Natural Science Foundation (7202153).

## Acknowledgments

We appreciate the patients and their families for participating in this study.

## Conflict of interest

The authors declare that the research was conducted in the absence of any commercial or financial relationships that could be construed as a potential conflict of interest.

## Publisher’s note

All claims expressed in this article are solely those of the authors and do not necessarily represent those of their affiliated organizations, or those of the publisher, the editors and the reviewers. Any product that may be evaluated in this article, or claim that may be made by its manufacturer, is not guaranteed or endorsed by the publisher.
