# Supplementary material for: Skeletal outcomes of patients with osteogenesis imperfecta during drug holiday of bisphosphonates: a real-world study
Source: Front Endocrinol (Lausanne). 2022 Sep 26;13:901925. doi: 10.3389/fendo.2022.901925 (PMC9549175; doi:10.3389/fendo.2022.901925)
Supplement: Supplementary file 2 [file Table_1.docx]

**Supplementary Table 1. Characteristics of a patient with seven years follow-up after BPs discontinuation**

|  | **0’** | **1^st^** | **2^nd^** | **3^rd^** | **4^th^** | **5^th^** | **6^th^** | **7^th^** |
| --- | --- | --- | --- | --- | --- | --- | --- | --- |
|  | **14 years** | **15 years** | **16years** | **17years** | **18 years** | **19years** | **20years** | **21years** |
| **Ca, mmol/l** | 2.32 | 2.13 | 2.42 | 2.49 | 2.38 | 2.41 | 2.43 | 2.33 |
| **P, mmol/l** | 1.13 | 1.24 | 1.3 | 1.23 | 1.08 | 1.05 | 1.27 | 1.24 |
| **25-hydroxy vitamin D, ng/ml** | - | 6.6 | - | - | 20.4 | 27.9 | 28.4 | 15.7 |
| **ALP, U/l** | 159 | 136 | 138 | 162 | 146 | 193 | 162 | 266 |
| **β-CTX, ng/ml** | 0.6 | 0.792 | - | 0.792 | 0.728 | 0.506 | 0.73 | 0.63 |
| **PTH, pg/ml** | - | 80.6 | 52 | - | - | 51.1 | 57 | 86.4 |
| **ALT, u/l** | 8 | 21 | 11 | 11 | 15 | 20 | 19 | 21 |
| **Cr, umol/l** | 47 | 51 | 63 | 69 | 64 | 74 | 70 | 62 |
| **Height, cm** | 160 | 162 | - | - | 162 | 165 | - | - |
| **Lumbar spine Areal BMD (g/cm2)** | 1.127 | - | - | 1.231 | 1.241 | 1.275 | - | 1.289 |
| **Lumbar spine Areal BMD Z-score** | 2.02 | - | - | 2.08 | 2.17 | - | - | - |
| **Femoral neck Areal BMD (g/cm2)** | 0.896 | - | - | 0.999 | 1.005 | 1.043 | - | 0.936 |
| **Femoral neck Areal BMD Z-score** | 0.04 | - | - | 0.12 | 0.18 | - | - | - |
| **Troch Areal BMD (g/cm2)** | 0.529 | - | - | 0.652 | 0.640 | 0.705 | - | 0.514 |
| **Total Hip Areal BMD (g/cm2)** | - | - | - | 0.975 | 0.998 | 1.045 | - | 0.917 |
| **Fracture** | Femoral | None | None | None | None | Femoral | None | Femoral |
| **Bone pain** | None | None | None | None | None | None | None | None |

-: Not available; BMD: bone mineral density; BPs: bisphosphonates; Ca: calcium; P: phosphate; 25OHD: 25-hydroxy vitamin D; ALP: alkaline phosphatase; β-CTX: β-cross-linked C-telopeptide of type I collagen; PTH: parathyroid hormone; Cr: creatinine.
